# Supplementary material for: Clinical epidemiology of COVID-19 among hospitalized children in rural western Kenya
Source: PLOS Glob Public Health. 2023 Jun 14;3(6):e0002011. doi: 10.1371/journal.pgph.0002011 (PMC10266603; doi:10.1371/journal.pgph.0002011)
Supplement: S1 Checklist — (DOCX) [file pgph.0002011.s003.docx]

STROBE Statement—checklist of items that should be included in reports of observational studies

|  | Item No | Recommendation | Page  No |
| --- | --- | --- | --- |
| **Title and abstract** | 1 | 1. Indicate the study’s design with a commonly used term in the title or the abstract   Clinical epidemiology of COVID-19 among hospitalized children in rural western Kenya: a cohort study | 1 |
|  |  | 1. Provide in the abstract an informative and balanced summary of what was done and what was found   We conducted a cohort study among 355 hospitalized Kenyan children and out of them 55 were positive for COVID-19. The commonest clinical features among COVID-19 cases were fever (42/55, 76%), cough (19/55, 35%), nausea and vomiting (19/55, 35%), and lethargy (19/55, 35%). Among positive participants, 8/55 (14.5%) died; Out of 49 tested, 9 (17%) had PCR-positive stool or rectal swabs but none had SARS-CoV-2 detected by culture. | 2 |
| Introduction | | | |
| Background/rationale | 2 | Explain the scientific background and rationale for the investigation being reported  In resource-limited settings, where children have a higher prevalence of comorbidities, including malnutrition, HIV and other infections, COVID-19 management maybe more complicate and may have worse clinical outcomes. However, there are limited data on the epidemiologic profile, diagnosis, and risk factors for COVID-19 among pediatric patients in resource-limited settings. The symptomatic overlap of COVID-19 with other prevalent diseases in resource-limited settings, including diarrheal disease, malaria, and pneumonia, may make the syndromic identification of COVID-19 challenging. Also, feco-oral transmission may be an important mode of transmission, particularly in low resource settings where access to adequate sanitation and clean water may be limited . | 3 |
| Objectives | 3 | State specific objectives, including any prespecified hypotheses  We sought to identify clinical and sociodemographic correlates of COVID-19 among hospitalized children in Kenya and to evaluate PCR and culture-based detection of SARS-CoV-2 in fecal samples as a measure of the potential for fecal transmission. | 4 |
| Methods | | | |
| Study design | 4 | Present key elements of study design early in the paper  A prospective cohort study of Kenyan children | 4 |
| Setting | 5 | Describe the setting, locations, and relevant dates, including periods of recruitment, exposure, follow-up, and data collection  We systematically tested all children admitted to three hospitals in Kenya (Homa Bay County Referral, Kisii Teaching and Referral, and Migori County Referral hospitals), who were aged two months to 15 years for SARs CoV-2 between March 1 and June 30, 2021. Baseline clinical and sociodemographic data were collected from all patients prior to SARs CoV-2 testing. Participants were scheduled for monthly follow-up visits in the outpatient clinics, unless the participant was hospitalized at the time of the visit. Follow-up ended when the child completed their six-month follow-up visit or when a child died. Families that missed the 6-month visit and were not traceable via phone calls or home visits were declared lost to follow up. Nasopharyngeal swab, rectal swab, stool, and blood samples were taken at admission. | 4 |
| Participants | 6 | (*a*) *Cohort study*—Give the eligibility criteria, and the sources and methods of selection of participants. Describe methods of follow-up  Confirmed cases were enrolled in a prospective cohort study of hospitalized children aged two months to 15 years if they planned to remain within the hospital catchment area for at least six months, and consented to participate in the study. Cohort participants were followed daily during hospitalization and monthly for 6-months after hospital discharge. | 4 |
|  |  |  |  |
| Variables | 7 | Clearly define all outcomes, exposures, predictors, potential confounders, and effect modifiers. Give diagnostic criteria, if applicable  The primary outcomes of this study were the clinical profile and correlates of SARS-CoV-2 infection and its detection by PCR and culture in stool or rectal swab samples. Secondary outcomes include the mortality rate among cohort participants.  The clinical variables were sickle cell disease, fever, lethargy, vomiting/nausea, difficulty of breathing, convulsion, diarrhea, cough, headache, not feeding, abdominal pain, fatigue, reduced air entry, wheeze, chest indrawing, crackles, capillary refill, activity (mental status of being irritable/Agitated or lethargic), and jaundice. The sociodemographic characteristics were age, sex, nutritional status (wasting measured by mid-upper arm circumference (MUAC), weight for height Z score (WHZ), and body mass index (BMI), and stunting), breastfeeding, birth order, caregiver marital status, and caregiver educational status. | 5 |
| Data sources/ measurement | 8* | For each variable of interest, give sources of data and details of methods of assessment (measurement). Describe comparability of assessment methods if there is more than one group  Baseline clinical and sociodemographic data were collected from all patients prior to SARs CoV-2 testing. Nasopharyngeal swab, rectal swab, stool, and blood samples were taken at admission. Cohort participants were followed daily during hospitalization and monthly for 6-months after hospital discharge. Participants were scheduled for monthly follow-up visits in the outpatient clinics, unless the participant was hospitalized at the time of the visit. When in-person visits were not possible, interviews were conducted through a phone call and participants' vital status was recorded. | *4* |
| Bias | 9 | Describe any efforts to address potential sources of bias  Only participants remained within the hospital catchment area for at least six months and consented to participate in the study were included. When in-person visits were not possible, interviews were conducted through a phone call and participants' vital status was recorded. Families that missed visits were traced via phone calls or home visits before declared lost to follow up. To reliably determine the isolation of the virus from fecal samples, the samples were re-inoculated in the same cell lines and incubated for an additional 10-12 days. | 4-5 |
| Study size | 10 | Explain how the study size was arrived at  Convenient sampling – we screened all admitted children over a 4 months period and included COVID-19 positive ones. | 4 |
| Quantitative variables | 11 | Explain how quantitative variables were handled in the analyses. If applicable, describe which groupings were chosen and why  Age – treated as a categorical variable to make more meaningful comparisons across groups | 5 |
| Statistical methods | 12 | 1. Describe all statistical methods, including those used to control for confounding   We used logistic regression to evaluate the association of each variable with SARS-CoV-2 infection. We fitted only unadjusted model due to lower sample size. | 5 |
|  |  | 1. Describe any methods used to examine subgroups and interactions   N/A |  |
|  |  | 1. Explain how missing data were addressed   N/A |  |
|  |  | (*d*) *Cohort study*—If applicable, explain how loss to follow-up was addressed  *N/A* |  |
|  |  | 1. Describe any sensitivity analyses   NA |  |

Continued on next page

| Results | | | |
| --- | --- | --- | --- |
| 6Participants | 13* | 1. Report numbers of individuals at each stage of study—eg numbers potentially eligible, examined for eligibility, confirmed eligible, included in the study, completing follow-up, and analysed   355 were examined and tested for COVID-19, and 55 positive cases were found and followed for 6 months. All participants with a positive or negative results were considered in the analysis | 6 |
|  |  | 1. Give reasons for non-participation at each stage   Out of 55 positve cases, 46 completed the follow-up – 8 died and one was untraceable. | 6 |
|  |  | 1. Consider use of a flow diagram   Inlcuded | 6 |
| Descriptive data | 14* | 1. Give characteristics of study participants (eg demographic, clinical, social) and information on exposures and potential confounders   A total of 355 children screened, and 55 (16%) were SARS CoV-2 positive, 296 (83%) tested negative for SARS CoV-2, and 4 (1%) had an inconclusive test result. Most participants were over two years old (216, 61%), a quarter (80, 23%) had severe wasting, and 6 (2%) were HIV infected. | 6 |
|  |  | 1. Indicate number of participants with missing data for each variable of interest   Included in table 1 | 7 |
|  |  | 1. *Cohort study*—Summarise follow-up time (eg, average and total amount)   *6 months* | 6 |
| Outcome data | 15* | *Cohort study*—Report numbers of outcome events or summary measures over time  8/55 (15%) of the participants died before the end of the 180-day follow-up period, with seven of the eight deaths occurring prior to hospital discharge (7/55 – 12.7% [95% CI: 5.3, 24.5%]). | *11* |
|  |  | *Case-control study—*Report numbers in each exposure category, or summary measures of exposure |  |
|  |  | *Cross-sectional study—*Report numbers of outcome events or summary measures |  |
| Main results | 16 | 1. Give unadjusted estimates and, if applicable, confounder-adjusted estimates and their precision (eg, 95% confidence interval). Make clear which confounders were adjusted for and why they were included   In the bivariable analysis, we observed a lower risk (marginally non-significant) of COVID-19 among males as compared to females (Crude Odds Ratio (COR): 0.59 (95%CI: 0.33, 1.05), p-value = 0.075) and among those who reported a headache compared to those who did not (COR: 0.34 (95%CI: 0.08, 0.98), p-value = 0.08) | 9 |
|  |  | 1. Report category boundaries when continuous variables were categorized   Age - <6months, 6-11 months, 11-23 months, 2-5 years, >=5 years | 9 |
|  |  | 1. If relevant, consider translating estimates of relative risk into absolute risk for a meaningful time period   N/A |  |
| Other analyses | 17 | Report other analyses done—eg analyses of subgroups and interactions, and sensitivity analyses  N/A |  |
| Discussion | | | |
| Key results | 18 | Summarise key results with reference to study objectives  In western Kenya, fever, cough, nausea and vomiting, and lethargy were the most frequent signs and symptoms observed among children testing positive for COVID-19. However, there was no difference in the sociodemographic and clinical characteristics of children with confirmed COVID-19 in comparison to those children who tested negative for COVID-19. This finding highlighting the challenges clinicians face in identifying children with COVID-19 in the absence of laboratory confirmatory testing, particularly given the overlap of these non-specific symptoms with other common illnesses frequently presenting to hospital | 13 |
| Limitations | 19 | Discuss limitations of the study, taking into account sources of potential bias or imprecision. Discuss both direction and magnitude of any potential bias  The study had a relatively small sample size and the observational nature of the study limits our ability to infer causality. Secondly, the study was conducted in a hospital setting and these results may not be generalizable to children living in the community. Finally, due to isolation protocols and a high rate of early mortality, we were unable collect whole stool or rectal swab samples on a small number of children, which may have introduced a degrees of selection bias. | 14-15 |
| Interpretation | 20 | Give a cautious overall interpretation of results considering objectives, limitations, multiplicity of analyses, results from similar studies, and other relevant evidence  The non-specific nature of the signs and symptoms associated with COVID-19 in children make it difficult to differentiate from other common pediatric infections in sub-Saharan Africa. This poses a particular challenge in resource-limited settings where diagnostics are often unavailable or unaffordable. Mortality among children hospitalized with COVID-19 was high but appears comparable to mortality observed among children hospitalized for other causes in the region. SARs CoV-2 was only detected in stool or rectal swabs by PCR in a quarter of children with confirmed COVID- 19 infection and live virus was not detected in any fecal samples. These data suggest that fecal transmission may not play a substantial role in the transmission of the virus. | 15 |
| Generalisability | 21 | Discuss the generalisability (external validity) of the study results  This study can be generalized to hospitalized children in sub-Saharan Africa | 15 |
| Other information | | | |
| Funding | 22 | Give the source of funding and the role of the funders for the present study and, if applicable, for the original study on which the present article is based  Bill & Melinda Gates Foundation [INV016894]. | 15 |
