# Supplementary material for: Clinical epidemiology of COVID-19 among hospitalized children in rural western Kenya
Source: PLOS Glob Public Health. 2023 Jun 14;3(6):e0002011. doi: 10.1371/journal.pgph.0002011 (PMC10266603; doi:10.1371/journal.pgph.0002011)
Supplement: Attachment — Submitted filename: Response to the reviewer.docx [file pgph.0002011.s004.docx]

Review Comments to the Author

Reviewer #1: Tsegaye et al. describe the clinical characteristics and associations with COVID-19 among hospitalized children near Kisumu, Kenya. This well-written manuscript represents multiple important contributions, including lack of culturable SARS-CoV-2 in stool from hospitalized children, and similar demographic and clinical characteristics in hospitalized children regardless of SARS-CoV-2 status.
A CONSORT diagram could help to show who was included and excluded from analysis for different reasons, and who was followed-up to ascertain survival.
It appears only a small fraction (53 out of >300) children were able to be followed-up to ascertain outcomes. Could the authors comment on potential biases due to the strong down-selection of who was followed-up?

**Response:**

Thanks so much for your supportive comments. The analysis has two broad sections. The first is about correlates of COVID-19 which included test-negative participants (300 children), and the second is on the outcome of test-positive individuals (55 children). Only 53 of the 300+ children were found to have COVID-19 status, and only COVID-19 patients were followed up to determine their outcome. We have revised the CONSORT diagram to depict the inclusion and exclusion procedure more clearly.

Old figure

New figure:
